# YB-1 expression promotes epithelial-to-mesenchymal transition in prostate cancer that is inhibited by a small molecule fisetin

**DOI:** 10.18632/oncotarget.1790

**Published:** 2014-02-19

**Authors:** Mohammad Imran Khan, Vaqar Mustafa Adhami, Rahul Kumar Lall, Mario Sechi, Dinesh C. Joshi, Omar M. Haidar, Deeba Nadeem Syed, Imtiaz Ahmad Siddiqui, Shing-Yan Chiu, Hasan Mukhtar

**Affiliations:** ^1^ Department of Dermatology, School of Medicine and Public Health, University of Wisconsin, Madison, WI; ^2^ Department of Neurophysiology, School of Medicine and Public Health, University of Wisconsin, Madison, WI; ^3^ Department of Chemistry and Pharmacy, University of Sassari, Italy

**Keywords:** Y-box potein-1, Fisetin, EMT, Prostate cancer, invasion, migration

## Abstract

Epithelial-to-mesenchymal transition (EMT) plays an important role in prostate cancer (PCa) metastasis. The transcription/translation regulatory Y-box binding protein-1 (YB-1) is known to be associated with cancer metastasis. We observed that YB-1 expression increased with tumor grade and showed an inverse relationship with E-cadherin in a human PCa tissue array. Forced YB-1 expression induced a mesenchymal morphology that was associated with down regulation of epithelial markers. Silencing of YB-1 reversed mesenchymal features and decreased cell proliferation, migration and invasion in PCa cells. YB-1 is activated directly via Akt mediated phosphorylation at Ser^102^ within the cold shock domain (CSD). We next identified fisetin as an inhibitor of YB-1 activation. Computational docking and molecular dynamics suggested that fisetin binds on the residues from β1 - β4 strands of CSD, hindering Akt's interaction with YB-1. Calculated free binding energy ranged from −11.9845 to −9.6273 kcal/mol. Plasmon Surface Resonance studies showed that fisetin binds to YB-1 with an affinity of approximately 35 μM, with both slow association and dissociation. Fisetin also inhibited EGF induced YB-1 phosphorylation and markers of EMT both in vitro and in vivo. Collectively our data suggest that YB-1 induces EMT in PCa and identify fisetin as an inhibitor of its activation.

## INTRODUCTION

Prostate cancer (PCa) is the leading cause of cancer related deaths among American men. When detected early the five year survival rate is close to 100% however, detection at advanced metastatic stages severely declines the overall survival [1]. These statistics suggest that high mortality in PCa patients is due to metastasis. Metastasis is a multistep process by which cancer cells disseminate from their primary site to form secondary tumors at a distant site. Metastasis involves a series of steps including local invasion, intravasation, transport, extravasation, and colonization.

During embryonic development cells travel long distances to reach their final destinations. To achieve this, epithelial cells rely on a very fine tuned and highly regulated program known as epithelial to mesenchymal transition (EMT) that converts them into a mesenchymal state. Cancer cells use this program to metastasize and colonize at distant sites [2]. Often the metastatic EMT resembles the molecular landscape of the developmental EMT such as loss of cell-cell junctions, apico-basal polarity and inheritance of migratory and invasive features. The EMT program is primarily orchestrated by one or several transcription factors (TF), mainly in response to variety of extracellular signals like EGF, FGF, IGF, TGF-β etc. [3]. The classical family of TF include Snail, Twist, Zeb, Slug etc.[4-6]. Recent research has added numerous new proteins than can direct the EMT program either directly or indirectly by inducing the classical TF. Among them Y-box–binding protein-1 (YB-1) is a recently identified protein known to induce EMT in different cancers [7].

YB-1 is a broad-specificity RNA-binding protein, mainly involved in regulation of mRNA transcription, splicing, translation, and stability [8]. Recent literature suggests its important role in a variety of cancers including prostate [9]. YB-1 plays various biologic roles in both the nucleus and the cytoplasm; however, as a transcription factor within the nucleus, YB-1 regulates the expression of several genes, including proliferating cell nuclear antigen, epidermal growth factor receptor (EGFR), DNA topoisomerase II, CD44, phosphotidylinositol-4,5-biphosphat 3-kinase, (PIK3CA,and MET) [10-14]. In PCa YB-1 is associated with cell growth, anti-apoptosis, and multi drug resistance [15-16]. YB-1 directly regulates androgen receptor transcription and was found to be upregulated during androgen ablated tumor progression in a mouse xenograft model, suggesting that YB-1 plays a role in the progression of both androgen-dependent and castrate resistant PCa [17]. YB-1 is well linked with Twist1 that regulates YB-1 transcription upstream in PCa [18]. Similarly YB-1 induces the expression of clusterin, an important EMT mediator [19], clearly suggesting some involvement of YB-1 in EMT during PCa progression. Based on existing literature it is clearly evident that YB-1 is associated with a variety of cancers including prostate and has been specifically projected as an interesting drug target.

Bioactive food components including plant polyphenols are known to have potent anticarcinogenic activities [20]. An important advantage with plant based polyphenols, especially those from dietary sources, is that they are perceived as non-toxic and have wide human acceptance [21]. Fisetin (3,7,3’,4’-tetrahydroxyflavone) belongs to such a flavonol subgroup of flavonoids along with quercetin, myricetin and kaempferol. It is present in many fruits and vegetables most notably strawberries, apples, persimmons, kiwis, cucumbers and onions. We have previously shown that, fisetin acts as a dual inhibitor of the PI3K/Akt and the mTOR pathways [22]. Also fisetin induces cell cycle arrest, inhibits androgen signalling and tumor growth in prostate cancer models [23-24].

In the current study we demonstrate that YB-1 expression leads to a change in cellular morphology towards a mesenchymal phenotype and induces migration and invasion of cells. We also identify a small molecule fisetin that interacts within the cold shock domain (CSD) domain of YB-1 and inhibits its phosphorylation at Ser^102^ both *in vitro* and in *in vivo*.

## RESULTS

### YB1 is overexpressed and inversely associated with E-cadherin in PCa

**Figure 1:**
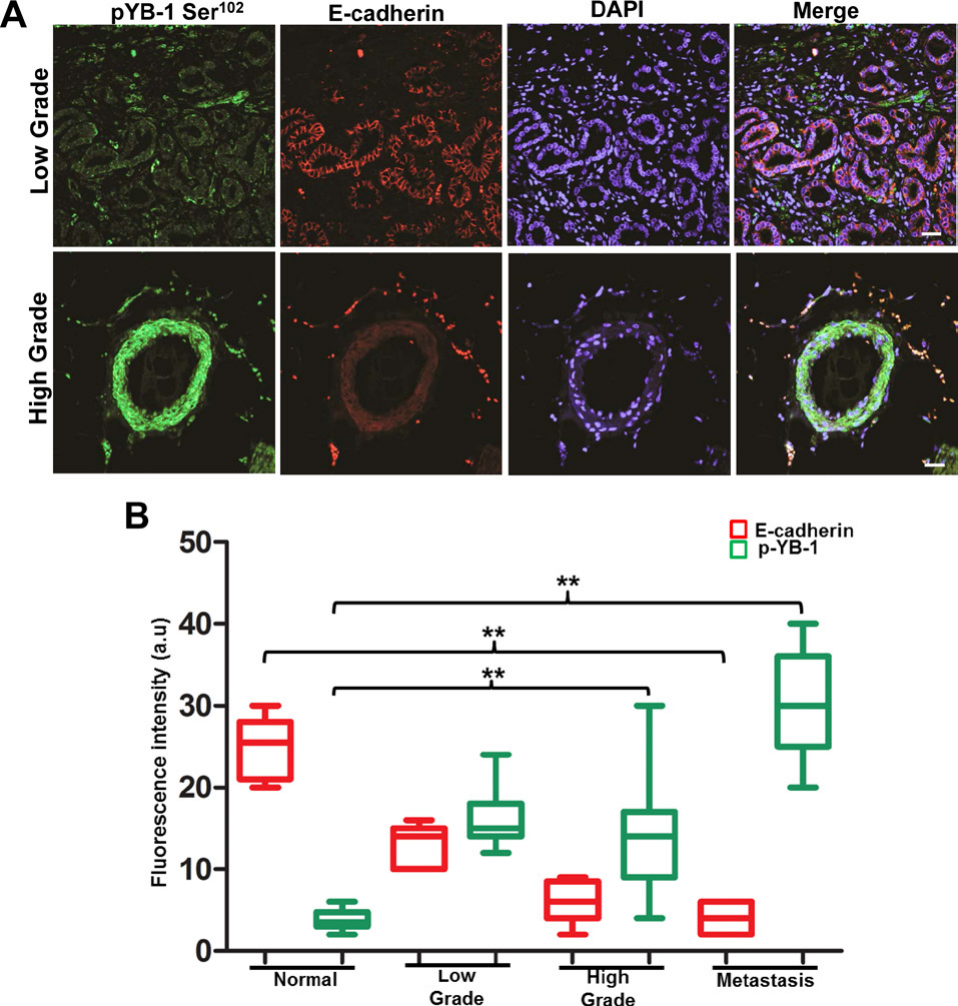
YB-1 expression increases with increase in tumor grade and is negatively correlated with E-cadherin expression in human PCa tissues Representative immunofluorescence images of human PCa tissues stained for YB-1 and E-cadherin. Human PCa tissue array containing a total of 42 cores; normal (n=8), low grade (n=7), high grade (n=21), and metastasis (n=6) was co-stained with anti pYB-1 (Green) and E-cadherin (Red) antibodies. Image acquisition settings were kept identical to ensure the comparability between individual groups. scale bar=50 μm (B) Box plot analysis of fluorescence intensity of images in A using the NIH image J software. p-values were calculated using GraphPad prism v5.0 as described. *p<0.05 and **p<0.01. Fluorescence intensities are represented as arbitrary units. DAPI was used as a nuclear staining control.

To determine whether YB-1 levels and localization correlate with Gleason grade in PCa, YB-1 staining was evaluated in a tissue array of benign, low (Gleason grade 2), intermediate (Gleason grade 3), and high grade (Gleason grade 4 or 5) PCa. In the benign prostate acini, YB-1 is expressed at a low level and is primarily localized in the cytoplasm of epithelial cells. Following transformation from benign to malignant, YB-1 expression levels are elevated and also demonstrate an increased expression in both peri-nuclear and nuclear spaces along with cytoplasmic localization (Supplementary Fig. 1). These results clearly suggest that YB-1 increases throughout malignant progression with increase in Gleason grade. Next, we compared the expression of E-cadherin in the same tissue samples by using dual labeling. Results showed significant inverse relation between E-cadherin and YB-1 in high grade prostate tissue samples when compared with low grade prostate tissue samples (Fig. 1A & B). Overall these results confirm an inverse relationship in E-cadherin/YB-1 ratio in high grade PCa tissues reinforcing a role for YB-1 in PCa.

### Endogenous EMT induces YB1 expression

**Figure 2:**
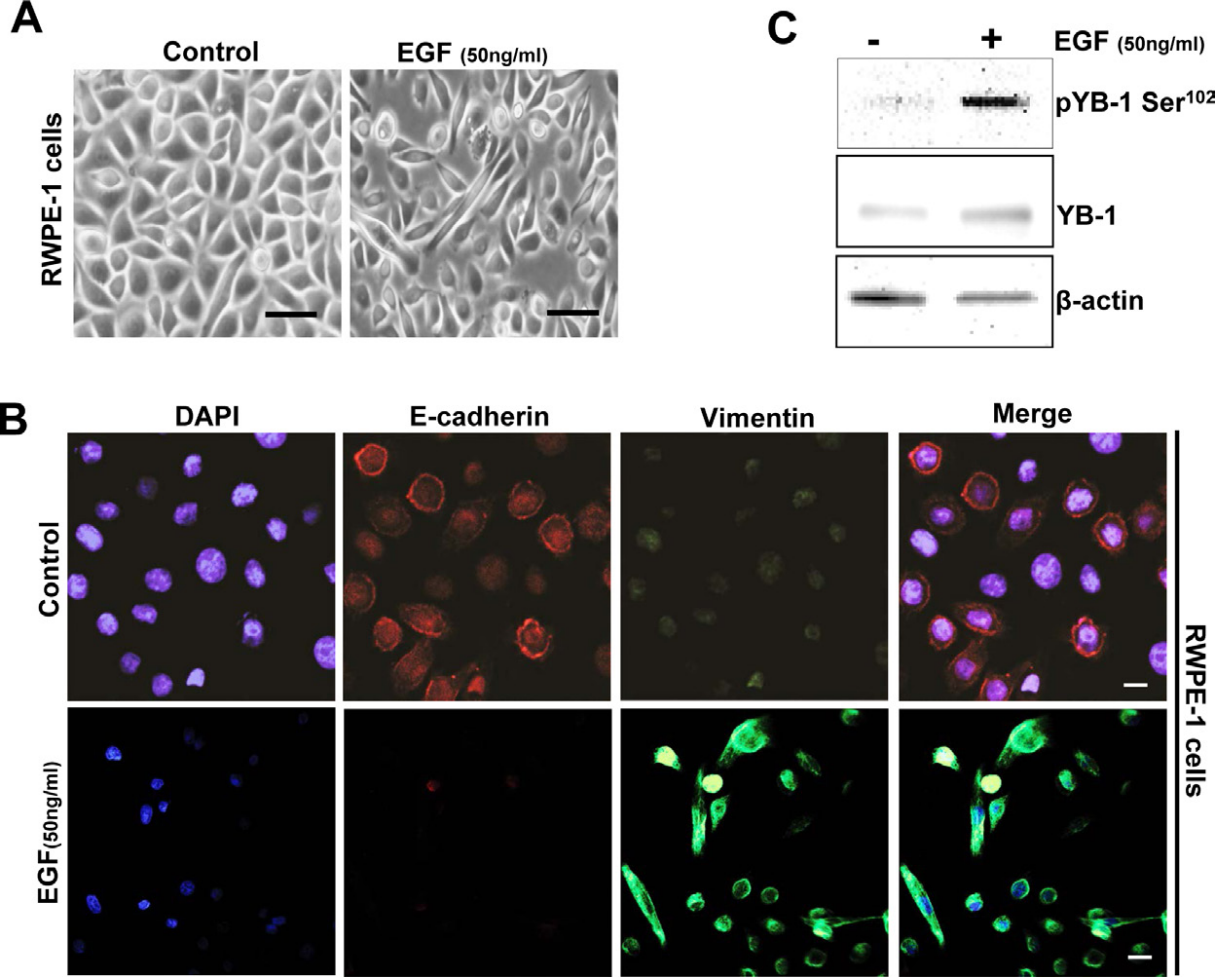
YB-1 Phosphorylation is increased in an endogenous EGF model of EMT (A) Representative phase contrast images showing EGF induced mesenchymal like morphology in cells. RWPE-1 cells were cultured in complete KSFM medium for 24h. Cells were starved for 4h and then treated with or without EGF (50ng/ml) for 48h. (B) Representative immunofluroscent images showing low E-cadherin and increased vimentin expression in EGF treated cells. Scale bar=50 μm. (C) Western blot analysis showing that pYB-1 Ser^102^ is induced in EGF treated cells. β-actin was used as a loading control.

Aberrant overexpression of EGFR has been associated with both hormone-refractory and metastatic PCa and is accompanied with proliferation, survival, invasion and metastasis. EGF, the predominant ligand to EGFR/ErbB1 receptors has been shown to induce EMT in a variety of epithelial cancers including prostate [25-26]. A recent study has also shown peculiar YB-1 phosphorylation along with activation of YB-1 interacting kinases including Akt, EGF and MEK in EGF treated PCa cell lines [27]. Taking these observations in consideration we sought to determine the expression and phosphorylation of YB-1 in an endogenous model of EMT using RWPE-1 cells. As anticipated we observed that exposure to EGF induced EMT like morphology in (Fig. 2A) that was associated with down regulation of E-cadherin and upregulation of vimentin as observed by immunofluorescence (Fig. 2B). Most importantly, in this model of EMT we observed YB-1 phosphorylation is significantly upregulated (Fig. 2C).These observations divulge a physiologically relevant role of YB-1 in endogenously induced EMT by EGF.

### YB-1 induces a mesenchymal phenotype in non-tumorigenic prostate epithelial cells

**Figure 3:**
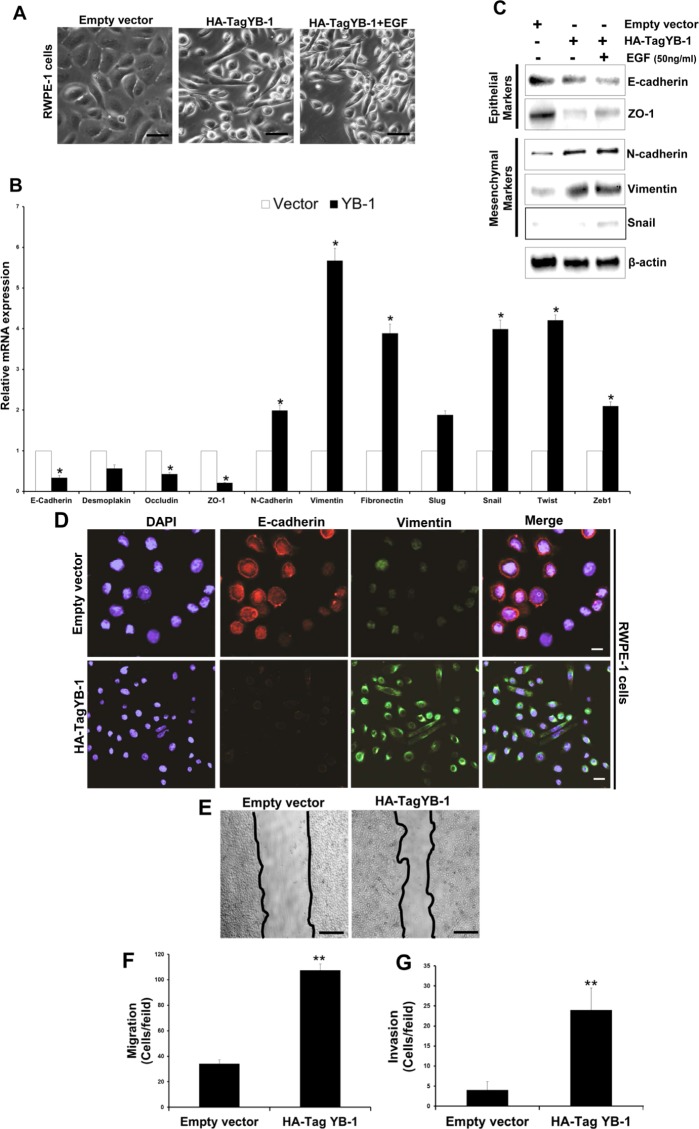
YB-1 induces EMT in non-tumorigenic prostate epithelial cells (A) Representative images showing the effect of forced expression of YB-1 on mesenchymal like changes in RWPE-1 cells. RWPE-1 cells were transfected with empty vector or YB-1 expression constructs, cultured in dishes and treated with EGF (50ng/ml). Scale bar = 50 μm. (B) Histogram showing the effect of YB-1 induced changes in transcript levels of various EMT related markers in RWPE-1 cells. Expression of various epithelial and mesenchymal markers was analyzed by qRT-PCR. Each transcript level from empty vector cells was set as 1; *p<0.05 (C) Representative blots showing expression of various epithelial and mesenchymal markers analyzed by western blotting in YB-1 transfected cells treated with or without EGF (50ng/ml) for 24hr. (D) Representative images showing immunofluroscence staining for E-cadherin (epithelial) and vimentin (mesenchymal) in vector or YB-1 transfected cells in presence or absence of EGF (50ng/ml) for 24hr.Scale bar = 50 μm (E) Representative images showing that YB-1 enhances the migration of RWPE-1 cells. Vector- or YB-1-expressing cells were plated on top of a transwell chamber in media without EGF for 2 days. Images show cell migration in a wound closure assay. (F & G) Histograms showing migration and invasion (mean ± SD) of cells with YB-1 overexpression without EGF. Mean cell numbers/field ± SD of experiments performed in triplicate is shown. **p<0.01.

We first determined the expression of both pYB-1 and YB-1 in non-tumorigenic and tumorigenic PCa cell lines. A higher level of YB-1 protein was observed in all tumorigenic PCa cells as compared to non-tumorigenic cells (Supplementary Fig. 2A). To elucidate whether YB-1 overexpression plays a role in the EMT in PCa we overexpressed YB-1 in non-tumorigenic RWPE-1 cells which mostly maintains an epithelial phenotype. Post transfected RWPE-1 cells showed approximately 4.3 fold increase in levels of YB-1 (Supplementary Fig. 2B), that was associated with morphological changes indicative of EMT (Fig. 3A). These cells grew as loose colonies of elongated cells compared to the cuboidal, compactly attached cells of control empty vector transfected cells. Similarly, we observed significant reduction in the transcript levels of epithelial markers viz. E-cadherin, desmoplakin, zona-occuldin-1 (ZO-1) and occludin along with induction in transcript levels of mesenchymal markers viz. N-cadherin, vimentin and fibronectin in the YB-1 overexpressed cells (Fig. 3B). Due to loss of cell viability (Supplementary Fig. 2C) and induction of EMT features it is difficult to sustain the growth of these cells *in vitro*. Addition of EGF (50 ng/ml) in YB-1 overexpressed cells significantly enhanced the viability and further sustained the EMT, suggesting the important role of extracellular growth/mitogenic signals in YB-1 mediated EMT (Fig. 3A). We observed significant reduction in epithelial markers and induction in the expression of mesenchymal markers that were further enhanced by EGF (Fig. 3C). We further confirmed the reduction in E-cadherin and induction in vimentin expression by immunofluroscence (Fig. 3D). We performed migration and invasion assays in the same gain-of-function model and found significant enhancement in migration and invasion of YB-1 overexpressed cells when compared with control (Fig. 3E, F & G). These results confirm YB-1 capability to induce EMT like changes and enhancement in cell migration and invasion rates in non-tumorigenic prostate cells which was further sustained by EGF, clearly suggesting the role of YB-1 in PCa disease progression and metastasis.

### Identification of Fisetin, a small molecule inhibitor of YB-1 phosphorylation

**Figure 4:**
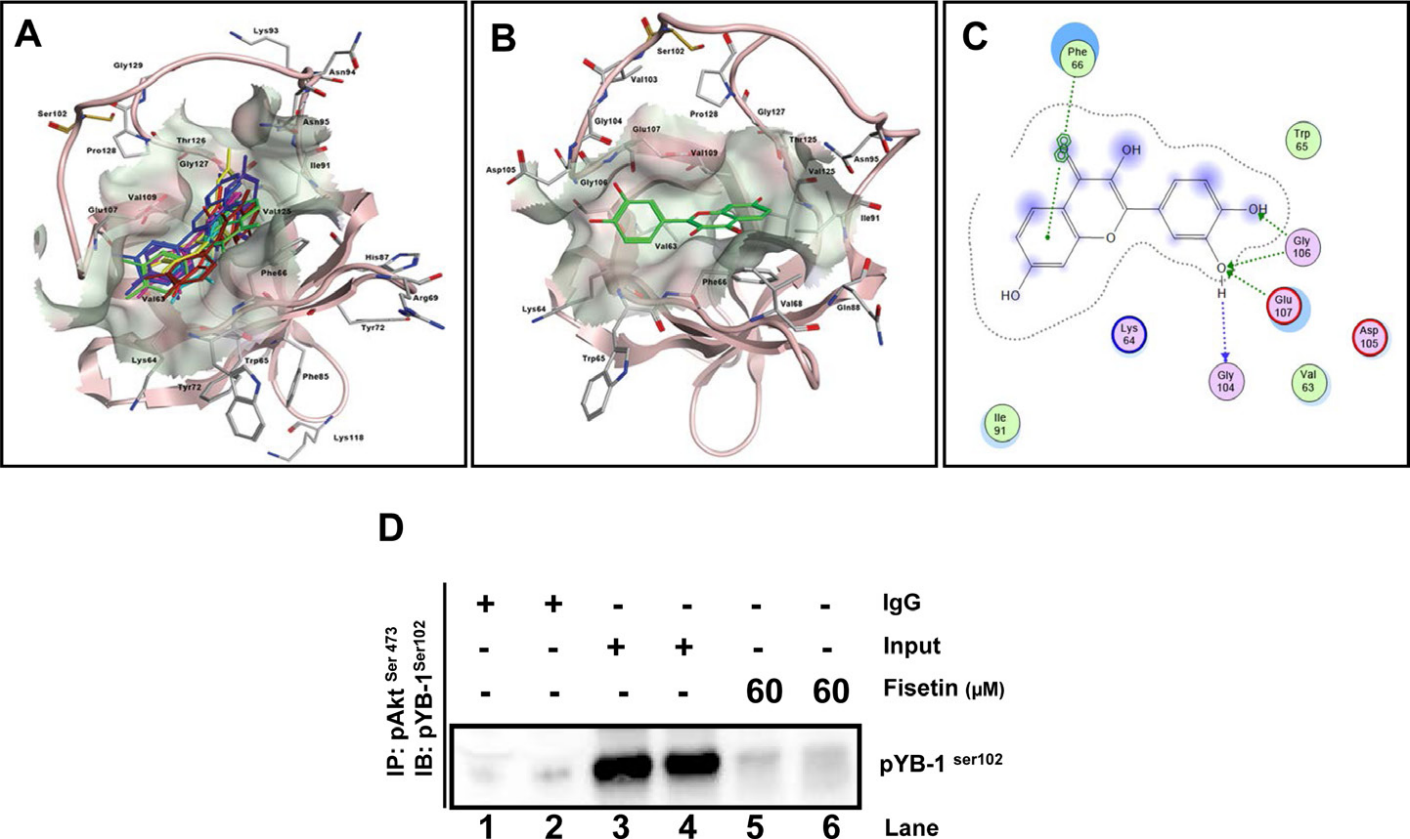
Fisetin binds to the CSD of YB-1 protein (A) In *silico* docking of fisetin within the putative binding site on CSD of YB-1. Top-ranked docking poses and representative view of the CSD domain. The protein is represented by cartoon, the binding site as molecular surfaces, and top-ranked energy poses as colored sticks. (B) Hypothetical disposition of the best energy fisetin docking pose within the CSD binding pocket of YB-1 protein. (C) Interaction of fisetin onto the CSD of YB-1 protein binding site. Amino acid residues (circles) are shown as follows: a) hydrophobic residues (green interior), b) polar residues (light purple), c) basic residues (blue ring), and d) acidic residues (red ring). Differences in solvent accessible surface area for fisetin ligand and for protein residues are plotted as a *blue* smud and a violet halo, respectively. The border of the binding pocket is marked by a dashed grey line. Dashed arrows denote H-bonds bonding (*blue* for H-bonds to the residue backbone, and *green* for H-bonds formed with the residue side chain), whereas the arrowheads indicate their direction (i.e., the donor is at the base of the arrow, and the acceptor is at the head). Green bis-arene rings within a dashed line denote possible arene-arene stacking. (D) Akt binds YB-1 *in-vitro.* LNCaP and PC3 cells were serum starved for 8h and treated with either EGF (50 ng/ml; lane 3 & 4) or EGF (50ng/ml) with fisetin (60 μM; lane 5&6) for 120 min then proteins from the cytoplasmic fraction were immunoprecipitated with the p-Akt^ser473^ antibody. Lane 1 and 2 shows IgG control for both LNCaP and PC3 cells respectively.

Current literature for YB-1 clearly suggests its pivotal role in cancer initiation, progression and drug resistance; however little attention has been paid to it as a direct therapeutic target. Akt, an established kinase is an important regulatory kinase for YB-1 activation through phosphorylation at Ser^102^. Additionally, mTOR upstream to Akt is also known to regulate YB-1 at translational level. We have shown that fisetin, a plant derived small molecule as a potent inhibitor of PI3K/Akt/mTOR pathway in different cancers including prostate. Based on the Akt/mTOR driven regulation of YB-1 we hypothesized that fisetin will inhibit Akt mediated YB-1 Ser^102^ phosphorylation. In order to assess this we initially performed molecular docking on the CSD of YB-1, known to be the most important domain for YB-1 Ser^102^ phosphorylation. Fisetin docked within the CSD on residues from β1 and β4 strands of YB-1 protein. Top-ranked energy scoring poses of fisetin share similar orientation patterns and binding modes as well as overlapping features on their hypothetical disposition within the binding site (Fig. 4A). Calculated free binding energy (ΔG_b_) for these conformations ranged from −11.9845 to −9.6273 kcal/mol, thus indicating good ligand-protein affinities. In particular, best energy docking conformation of fisetin showed consistent binding modes and tight affinity within the amino acid pocket that includes the following residues: Val63, Lys64, Trp65, Phe66, Ile91, Gly104, Asp105, Gly106, Glu107 (Fig. 4B). The most favorable conformation places the catecholic moiety in close proximity to the Glu107 and the Glys104 and106, involving H-bond interactions with these residues, whereas an arene-arene stacking between the phenyl backbone of the chromen-4-one ring system and the Phe66 was observed (Fig. 4C). The other amino acid residues located near the binding site establish van der Waals and hydrophobic interactions with fisetin. Interestingly, the residue Trp65, considered to be very important for the activity of YB-1, is also contained in the predicted binding pocket.

To estimate more reliable conditions of enzyme-ligand complex in time-dependent manner, further explorative MD simulations were performed. The analyses revealed a consistent shifting of the flexible loop constituted by the sequence from Tyr^99^ to Val^109^. In particular, in the course of the experiment, the Ser^102^ establishes molecular interactions with Glu^107^, also involving other residues such as Gly^104^ (Supplementary Fig. 3C), Tyr^99^ and Arg^101^ (Supplementary Fig. 3D & F). On the other hand, although several dynamic conformational changes of the protein were found, fisetin engaged in significant H-bond interaction with one catecholic hydroxyl and the Glu^107^, as well as Gly^104^ and Tyr^99^ (Supplementary Fig. 3C & F) during the simulation. Fisetin always docked within the predicted site thus promoting a stable ternary ligand-Glu^107^-Ser^102^ complex. In this scenario, we hypothesized that fisetin could be involved in stabilizing the Ser^102^-Glu^107^ interaction, thus affecting the function of Ser^102^. Interference with phosphorylation of YB-1 can indirectly affect the binding to DNA, thus preventing the function of transcription factor at a later stage. In order to confirm the modelling data we performed Plasmon Surface Resonance (PSR) and found that fisetin binds to YB-1 with an affinity of approximately 35 μM, with both slow association and dissociation (data not shown).

We next performed immunoprecipitation experiments to see the effect of fisetin on YB-1/Akt interaction and observed significant loss in the interaction that confirmed our molecular docking experiments (Fig. 4D). The above finding provided evidence that fisetin binds within the CSD region that might interfere with its binding with activating kinases and we confirmed this view by showing low interaction between Akt and YB-1.

### Fisetin inhibits EGF induced YB-1 phosphorylation and EMT in PCa cells

**Figure 5:**
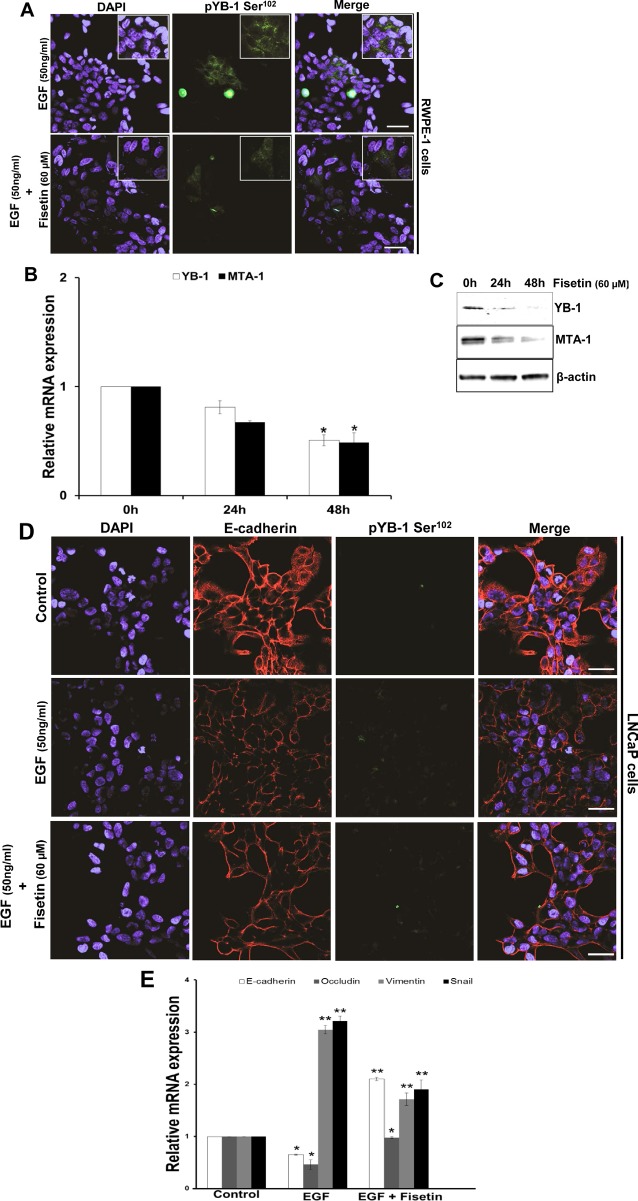
Fisetin inhibits EGF induced YB-1 phosphorylation and MTA-1 expression (A) Representative immunofluorescent images of LNCaP cells treated with EGF and fisetin and stained for pYB-1 Ser^102^. Cells were serum starved for 24h and then treated with either EGF (50 ng/ml) alone or EGF (50 ng/ml) + fisetin (60 μM) for 120 min and processed for Immunofluroscent staining of pYB-1 Ser^102^. (B) Histograms represent relative YB-1 and MTA-1 mRNA expression (mean±s.d). LNCaP cells were treated as above and YB-1 and MTA-1 gene expression measured at 24 & 48h by qPCR. *p<0.05. (C) Immunoblot images of LNCaP cells treated as above and MTA-1 protein expression was measured at 24 & 48h by western blotting. (D) Representative immunofluorescent images of LNCaP cells serum starved for 24h and then treated with either with EGF (50ng/ml) alone or EGF (50 ng/ml) + fisetin (60 μM) for 48 h and stained for E-cadherin and vimentin. Scale bar in A and D=50 μm. (E) Histogram represents relative mRNA expression of EMT genes by qPCR. LNCaP cells treated as stated in D and further qRT-PCR analysis was done to quantify various EMT genes. *p<0.05, **p<0.01.

Based on our data that pYB-1/YB-1 ratio is increased in the tumorigenic PCa cell lines, we next suppressed YB-1 expression by siRNA mediated gene silencing. As expected we observed significant reversal in cellular morphology towards an epithelial phenotype, and decrease in migration in the two PCa cell lines DU145 and C4-2 (Supplementary Fig. 4A & B). We next tested the efficacy of fisetin on decreasing the phosphorylation of YB-1 expression in presence of EGF in another PCa cell line LNCaP. As shown in Fig. 5A fisetin (60μM; 24h) treatment significantly reduced the EGF induced YB-1 phosphorylation. We also tested the effect of fisetin on YB-1 transcript levels and observed significant reduction at 48 h. Down regulation of MTA-1, a downstream target of YB-1 [28-30] further confirmed the effect of fisetin on YB-1. (Fig. 5B & C).

Next, we tested fisetin to inhibit the EMT in an EGF induced model of LNCaP cells. Cells treated with EGF exhibited decrease in epithelial makers E-cadherin and occuludin and increase in mesenchymal markers vimentin and snail. However, fisetin treatment resulted in significant recovery in the mRNA levels of epithelial marker E-cadherin but not occuludin. Similarly, fisetin treatment also down regulated the mRNA levels of vimentin and slug (Fig. 5D). In order to confirm the fisetin mediated inhibition of EMT at protein levels, we performed immunofluroscence staining of E-cadherin and vimentin in control, EGF, and EGF plus fisetin treated cells. As show in Fig. 5E control LNCaP cells showed strong E-cadherin expression and very low vimentin expression. However, EGF treatment decreased E-cadherin and enhanced vimentin fluorescence in LNCaP cells. Post EGF, treatment with fisetin (60μM; 24h) significantly recovered E-cadherin and suppressed the EGF induced vimentin expression. These results confirmed the potential of fisetin to inhibit the growth factor induced YB-1 phosphorylation and EMT in PCa cells.

### Fisetin treatment inhibits EMT *in vivo*

**Figure 6:**
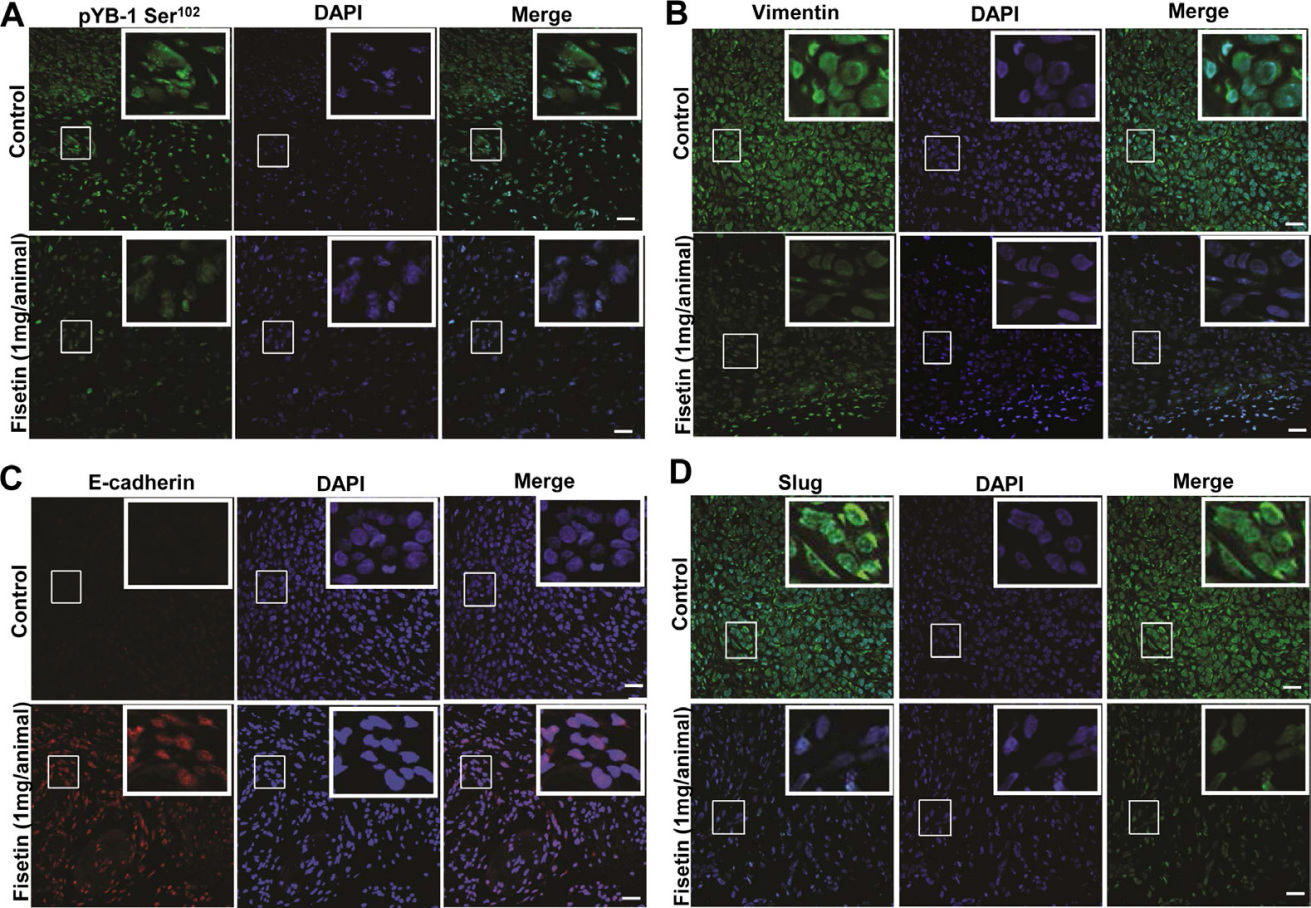
Fisetin treatment inhibits YB-1 phosphorylation and EMT *in vivo* (A) Representative immunofluorescent confocal images showing the expression of p-YB-1 Ser^102^ in tumor tissues from control and fisetin treated mice. Athymic nude mice were implanted with tumorigenic NB26 cells as described in the materials and methods section. Tumor tissues were harvested and processed for pYB-1 staining. (B) Representative immunofluorescent confocal images showing the expression of E-cadherin and vimentin. As described above tissues from the same cohort were stained for both E-cadherin and Vimentin. Images were captured by a Nikon Confocal microscope using the same settings as described in materials and methods. Scale bar=50 μm. DAPI was used as a nuclear staining control.

The *in-vitro* results prompted us to look forward for the similar effects in an *in vivo* scenario. We used nude mice implanted with the advanced PCa cell line NB26, known to be highly tumorigenic. Following the initial tumor growth the mice were given fisetin (1 mg/kg/per mice) for 28 days i.p. Post 28 days of fisetin treatment the mice were euthanized and tumors were harvested. Fisetin treatment significantly reduced both the tumor size and weight in the xenograft mice (data not shown), which corroborated well with our earlier findings showing fisetin as potent inhibitor of PCa progression (Khan et al.). We then performed immunofluroscence staining to assess the effect of fisetin on YB-1 phosphorylation and EMT related markers in the xenograft samples. We observed significant induction in the YB-1 phosphorylation in the non-treated tumor samples (Fig. 6A). Detailed analysis showed that pYB-1 Ser^102^ was localized in both cytoplasmic and nuclear compartments confirming earlier reports. We also observed loss of the epithelial marker E-cadherin and induction of mesenchymal markers vimentin and slug (Fig. 6B & C). Additionally, we also found significant induction of an important transcription factor slug known to be a target for YB-1 (Fig. 6D). These results confirmed that YB-1 phosphorylation is increased in PCa and also had a negative correlation with E-cadherin loss clearly corroborating the human PCa tissue immunofluorescence data. Next we stained the fisetin treated tumor samples for pYB-1 and observed significant decrease in the expression of pYB-1 both in cytoplasmic and nuclear compartments, clearly suggesting YB-1 as a target for fisetin (Fig. 6A). Further, we also checked the expression of EMT markers in these samples. As expected we observed significant induction in E–cadherin expression with concomitant reduction in vimentin (Fig. 6B & C). E cadherin is also known to be expressed in other cellular compartments and negatively influences processes like EMT and tumorigenesis. Fisetin induces E–cadherin expression not only in the membranal but also in other compartments, suggesting strong effect of fisetin on E-cadherin re-expression (Fig. 6B). These data clearly suggest that fisetin significantly reduces YB-1 phosphorylation and EMT.

### Fisetin inhibits TGF-beta induced YB1 expression and EMT in PCa cells

**Figure 7:**
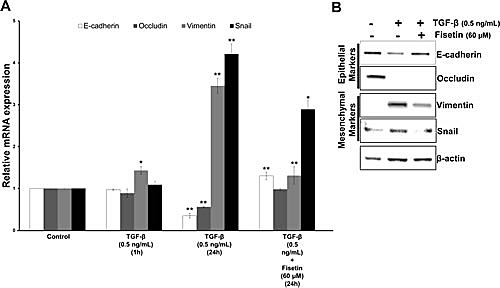
Fisetin inhibits TGF-β induced EMT in PCa cells (A) Histograms represent relative mRNA expression (mean±s.d) of EMT related genes. LNCaP cells were treated with 0.5 ng/mL of TGF-β for the indicated durations and qRT-PCR was conducted using the specific primer pairs and probes for EMT related genes. Each transcript level from nonrelated cells was set as 1. *p<0.05, **p<0.01 (compared with control). (B) Immunoblot images showing expression of EMT related proteins. LNCaP cells were treated as described in above for 24 h in the presence of fisetin (60 μM) and whole-cell extracts were made and analyzed by Western blot analysis with specified antibodies. β-actin was used as the loading control.

TGF-β induced EMT is a well known model to identify novel compounds/small molecules as inhibitors. To strengthen our data that fisetin is a potent EMT inhibitor we tested its efficay in the TGF-β induced EMT model. As presented in Fig. 7A TGF-β exposure for 1h did not induce any significant effect on both epithelial and mesenchymal mRNA transcript levels in PCa cell line (22Rv1); however, 24h treatment of TGF-β significantly reduced the mRNA transcripts of epithelial markers i.e. E-cadherin and occuludin, and also subsequently increased the mRNA transcripts of mesenchymal markers i.e. vimentin and snail. Similar results were observed at protein levels. Fisetin treatment significantly reversed the effect of TGF-β, as Fig. 7B clearly shows concomitant upregulation of E-cadherin and down regulation of mesenchymal markers, vimentin and snail. No change in occludin expression was observed at protein levels by fisetin, suggesting additional mechanisms. These results confirmed that fisetin inhibits EMT process in a relevant model.

## DISCUSSION

The major findings of this study are: (i) Forced overexpression of YB-1 in presence of EGF induces persistent EMT in non-tumorigenic prostate cells, (ii) YB-1 phosphorylation is strongly induced by EGF in prostate cells, (iii) YB-1 expression is inversely correlated with E-cadherin in human PCa samples, (iv) fisetin interacts within the CSD domain of YB-1 resulting in reduction in YB-1 phosphorylation at ser^102^ by reducing its interaction with activating Akt kinase and (v) fisetin inhibits EMT in both xenograft and in well-established *in vitro* TGF-β model suggesting its potential as an EMT inhibitor in PCa.

YB-1 overexpression in non-tumorigenic prostate cells (RWPE-1) induced EMT like features; however these cells tend to lose the phenotype and also showed significant growth retardation. We believe there could be two possible explanations for this. It may be possible that these cells were not supported by extracellular growth signaling/factors like EGF, IGF, bFGF etc. known to phosphorylate YB-1, which seems to be important for its oncogenic function and EMT inducing properties. This notion is well supported by previous studies that showed that in the absence of activated PI3K-Akt, YB-1 functions as a tumor suppressor mainly by inhibiting the cap-dependent translation [31]. Also, under similar conditions YB-1 showed growth suppressive properties [32]. Therefore, it can be speculated that YB-1 overexpression alone is not enough to induce a mesenchymal phenotype in non-tumorigenic epithelial cells. From our observations it seems that YB-1 mediated EMT program is a two tier mechanism, where YB-1 phosphorylation is enhanced by EGF and YB-1 further enhances the transcription and translation of vimentin and snail to confer mesenchymal like changes in the cells. However, it remains unclear whether the reduction of E-cadherin as observed in our results is a direct effect of YB-1 on its transcription and/or translation. It is possible that upregulation of snail, a potent and established negative regulator of E-cadherin by YB-1 might be responsible for this, an argument that needs further validation [7, 33-36]. The increased migration rate of YB-1 overexpressed cells is in agreement with other previous studies [26], however one striking observation found in our study was that overexpressed cells showed significantly enhanced migration rate even in the absence of EGF, suggesting that YB-1 controls this property of cells without getting phosphorylated by upstream kinases. The enhanced migratory rate could also be supported by matrix degrading metalloproteases's (MMP) known to be directly upregulated by YB-1 known to be involved in cellular migration [37-39].

EGF-induced EMT like morphology has been demonstrated as a crucial mechanism for the acquisition of invasiveness. EGF activates distinct signaling pathways, including ERK1/2, Akt, and Wnt to suppress the expression of epithelial proteins like E-cadherin and to increase the expression of mesenchymal proteins like vimentin. Recently EGF exposure to tumorigenic PCa cells was found to induce YB-1 phosphorylation in a dose and time dependent manner suggesting this signaling is a major effector of YB-1 mediated oncogenic function. Also YB-1 is known to regulate the expression of EGFR. We similarly observed that EGF exposure to non-tumorigenic PCa cells significantly enhanced YB-1 phosphorylation suggesting that EGF might utilize YB-1 to promote EMT and metastasis. This observation seems to be quite significant as activation of EGF-EGFR pathway has been associated with aggressiveness in PCa patients.

After ascertaining the involvement of YB-1 during PCa EMT, we next focused on the identification of a possible YB-1 inhibitor. We chose to test fisetin for a number of reasons: (a) fisetin is a dual inhibitor of both PI3K/Akt pathways [22], which is one of the major kinase for YB-1 phosphorylation, (b) fisetin inhibits mTOR pathway [23-24], which is now shown to be important for YB-1 mRNA translation [40] and based on our previous observations fisetin inhibits the growth of PCa. As observed in molecular docking experiments fisetin directly interacted within the CSD of YB-1 protein, important for binding of kinases and to phosphorylate and activate YB-1. Fisetin not only binds within the CSD of YB-1 but also alters its topology. These results suggest that fisetin inhibits Akt mediated YB-1 phosphorylation mainly by interfering with its binding to the CSD. Elevated Akt activity in the cell did not affect expression levels of YB-1, its subcellular localization, or general RNA-binding ability. Instead, phosphorylated YB-1 was less capable of cross-linking to the mRNA cap structure and of inhibiting cap-dependent translation of a reporter mRNA. These data suggest that YB-1 phosphorylation by Akt weakens its cap-binding capability; thereby facilitating translational activation of silenced mRNA species which mainly includes EMT related mRNA. Similar observations were made by [41] Law et al. 2010, using a small cell permeable peptide that binds within the flexible region and interferes with the 90 kDa ribosomal S6 kinase (RSK) binding with YB-1. We believe fisetin has several advantages over other strategies to inhibit YB-1. Fisetin not only decreases YB-1 phosphorylation but also affects the labile pool of both cytosolic and nuclear YB-1. It also acts on the upstream kinases PI3K/Akt/mTOR to regulate the transcriptional and translational of YB-1. Finally, fisetin is a naturally occurring plant based flavonoid with no documented toxicity *in vivo* and is readily available.

Based on these results we next tested fisetin to inhibit EMT in a widely accepted model of EMT induced by EGF and TGF-β. In PCa cells EGF induced the disruption of epithelial cell plasticity, upregulated snail, enhanced motility and invasion mainly through Akt-dependent mechanisms, eventually promoting EMT and invasiveness [38].Fisetin reduces EGF induced YB-1 phosphorylation and reverses EMT, suggesting fisetin's EMT inhibiting potential. We also found that fisetin significantly reverses the TGF-β induced EMT in PCa cells. Whether TGF-β induces YB-1 phosphorylation and is important for EMT induction in PCa cells remains to be elucidated. It seems plausible that fisetin inhibits mTOR that is known to be upregulated by TGF-β and plays an indispensable role in TGF-β mediated EMT [42-43].

In summary, we provide evidence that YB-1 is upregulated in human PCa, has an inverse relationship with epithelial marker E-cadherin and its forced expression in non-tumorigenic PCa cells induces EMT and enhances cellular migration. We also provide evidence that a small molecule fisetin physically interacts with YB-1 and inhibits its activity and subsequently inhibits EMT both *in vitro* and *in vivo.*

## METHODS

### Cell culture

The following human non-tumorigenic and tumorigenic cell lines were obtained from ATCC (Manassas, VA): RWPE-1, LNCaP, DU145, 22Rν1, PC-3, NB26 cells. Cell lines were characterized and authenticated by ATCC using a comprehensive database of short tandem repeat DNA profiles and maintained as per the recommendations of ATCC.

### Plasmids

Full-length pcDNA-HATagYB-1, pcDNA (empty vector), FLAG-YB-1 and FLAG-empty vectors were obtained from Prof. Peter Mertens (Aachen, Germany) and Prof. Sandra E. Dunn (Vancouver, Canada).

### Cell transfections

For plasmid transfections, 1×10^6^ cells (RWPE-1) were transfected with either 1 μg of pcDNA (empty vector) or pcDNA-HATagYB-1 vectors using Nucleofactor technology, Lonza (Anaheim, CA). After 6 hours medium was changed, cells were scraped 48hours for qPCR analysis and 72 hours for immunoblot analysis.

### siRNA

ON-TARGETplus SMARTpool siRNA for YB-1 and a nontargeting control siRNA were purchased from Dharmacon. Cells were incubated with siRNA (100 nM) and DharmaFECT reagent (#1) in antibiotic-free medium for 48 hours was used to transfect the cells.

### Real-time qPCR analysis for mRNA expression

Briefly, RNA was extracted from the non-tumorigenic, tumorigenic and tumor tissue samples using Rneasy kit (Qiagen), and reverse transcribed with iScript Reverse transcription supermix kit (Biorad). cDNA (1-100ng) was amplified in triplicate using gene specific primers (Table-1). Threshold cycle (*C*_*T*_) values obtained from the instrument's software were used to calculate the fold change of the respective mRNAs. Δ*C*_*T*_ was calculated by subtracting the *C*_*T*_ value of the housekeeping gene from that of the mRNA of interest. ΔΔ*C*_*T*_ for each mRNA was then calculated by subtracting the *C*_*T*_ value of the control from the experimental value. Fold change was calculated by the formula 2^−ΔΔ^^*CT*^.

### Cell viability assay

The effect of YB-1 overexpression (RWPE-1) and knockdown (DU145 and C4-2) on cell viability was determined by 3-[4,5-dimethylthiazol-2-yl]- 2,5-diphenyl tetrazolium bromide (MTT) assay. The absorbance was measured at 560 nm on a microplate reader (Bio-TEK Instruments).

### Cell migration and invasion assays

Cell migration and invasion was performed by using the commercially available kits from Millipore (Billerica, MA) as per the manufacturer protocol. However, for non-tumorigenic RWPE-1 cells KSFM complete medium with serum and for tumorigenic DU145 and C4-2 cells RPMI-1640 supplemented with 10% FBS was added to the lower chambers. Experiments were conducted in triplicate for each cell line.

### Immunofluorescence analysis

4-8 μm thick sections were obtained from frozen blocks of tumor xenograft tissues, and mounted on superfrost plus slides. Slides were stored at −80°C until needed. Prior to staining procedure, slides were warmed at room temperature for 5 min and fixed in ice-cold acetone for 15 min at 4°C, followed by washing in PBS-Tween 20 (x% PBST). LNCaP cells were fixed with 2% paraformaldehyde and washed 3 times with ice cold PBS. Further both types of slides were then blocked with normal serum block (mention serum name and % of serum here. Ex. Normal goat serum etc) followed by incubation with different antibodies with appropriate dilutions as described in supplementary table 1. After rinsing in PBST, slides were incubated with fluorescent-conjugated secondary antibody at 1:500 dilution in blocking buffer. Slides were then rinsed in PBST, and sections were mounted with ProLong Gold Antifade reagent containing DAPI (Invitrogen) and left in dark overnight. Fluorescence imaging was performed using Nikon A1 confocal microscope ((Nikon Instruments Inc. New York, USA). Images were acquired with 20x objective at 1024 × 1024 resolutions. Image analysis was accomplished using the Nikon Elements software.

### In silico molecular dynamics study

Ligand docking studies were performed using Molecular Operating Environment (MOE). Three-dimensional structure of cold-shock domain (CSD) of Y-box binding protein-1 (YB-1) used for docking experiments was downloaded from the PDB Data Bank (http://www.rcsb.org/-PDB code:1h95). Before docking, the protein structure was properly protonated using the Protonate 3D option, and was geometrically optimized and minimized by employing AMBER99 force field with a generalized Born solvation model. The partial charges were automatically calculated. The structure of fisetin was built in MOE and minimized before the docking, using the MMFF94x force field, with the systematic algorithms until a RMSD gradient of 0.001 kcal mol-1 Å-1 was reached. Rigid receptor-flexible ligand docking calculations were performed using the docking simulation feature MOE-dock by setting grid sizes that included the entire macromolecule. The triangle matcher was used as placement method to generate docking poses, and the London ΔGbinding scoring function that estimates the free energy of binding (kcal/mol) was used to rank hit candidates, after a force field based refinement and rescoring. Only the best scored poses generated in the docking experiments were retained and examined with MOE. After docking, a molecular dynamics (MD) simulation to the best docking complex was performed using the MD option (MOE-dynamic) included in the MOE suite of programs. Before MD experiment, a minimization energy calculation of the ligand-protein complex was conducted by using the above-mentioned procedure, until RMSD gradient 0.1. The other parameters were adjusted to default values, and the Nosé-Poincaré-Andersen thermostat algorithm in a canonical number of particles, volume, and temperature (NVT ensemble), was used for creating ensemble trajectory. The temperature was raised from 0 to 300 K, while the other parameters were kept as default. After system equilibration, 300 ps of MD were recorded and six snapshots were considered (50, 100, 150, 200, 250, and 300 ps).

### *In vivo* tumor xenograft model

Athymic (*nu/nu*) male nude mice (Harlan, USA) were housed under pathogen-free conditions with a 12-h light/12-h dark schedule and fed with an autoclaved diet *ad libitum*. We chose AR-positive NB26 cells for determining the *in vivo* effects of fisetin based on the fact that these cells form rapid and reproducible tumors in nude mice and also secrete PSA in the bloodstream of the host. Sixteen animals were then randomly divided into two groups, with 8 animals in group 1 and 8 animals in group 2. The first group of animals received i.p. injection of DMSO (30 μL) and served as control. The animals of groups 2 received i.p. injection of fisetin (1 mg/animal) in 30 μL of DMSO twice weekly. Tumor sizes were measured twice weekly as described previously^23^. All animals of group 1 and group 2 were sacrificed when tumors reached a volume of 1,200 mm^3^ in the control group. All procedures conducted were in accordance with the guidelines for the use and care of laboratory animals.

### Statistical analysis

Data were analyzed using GraphPad Prism (version 5; GraphPad Software). Two-tailed, unpaired *t* test was used. Data points in graphs represent mean ± SD, and *p* values < 0.05 were considered significant.

## SUPPLEMENTARY FIGURES AND TABLE




