# Supplementary material for: YB-1 expression promotes epithelial-to-mesenchymal transition in prostate cancer that is inhibited by a small molecule fisetin
Source: Oncotarget. 2014 Feb 19;5(9):2462–74. doi: 10.18632/oncotarget.1790 (PMC4058019; doi:10.18632/oncotarget.1790)
Supplement: Supplementary file 1 [file oncotarget-05-2462-s001.pdf]

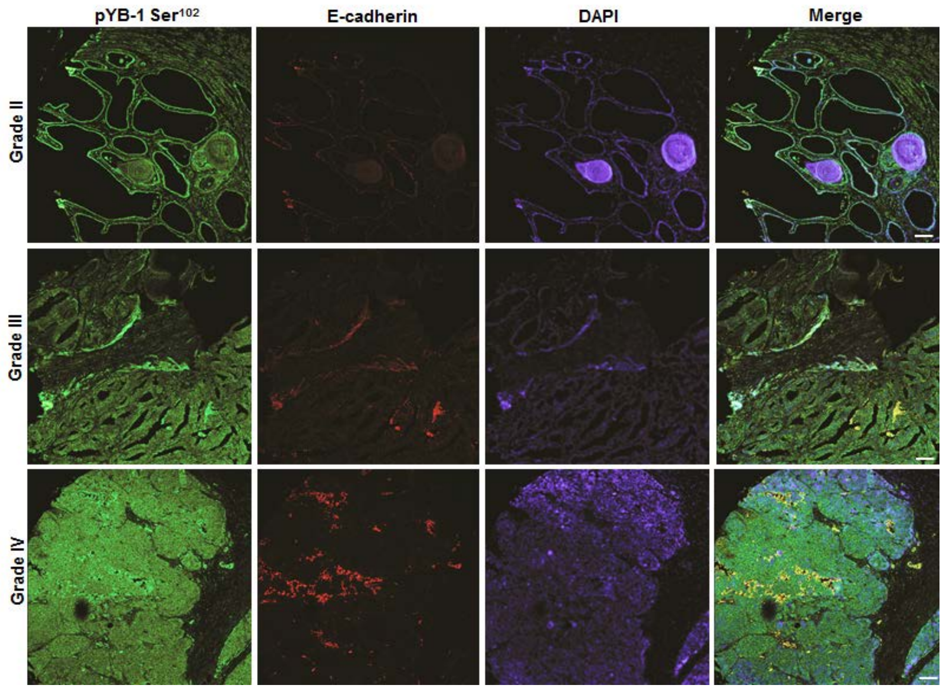

Supplementary Figure 1. Co-localization of pYB-1^{ser102} and E-cadherin in PCa tissues. Representative photomicrographs showing the co-localization of pYB-1^{Ser102} with the epithelial marker E-cadherin in human PCa tissues of various grades as assessed by an immunofluorescence assay. The merged lane shows the co-localization of 2 proteins. DAPI was used as a nuclear staining control. The images were captured using the same settings as described in figure 1. Scale bar =100µm.

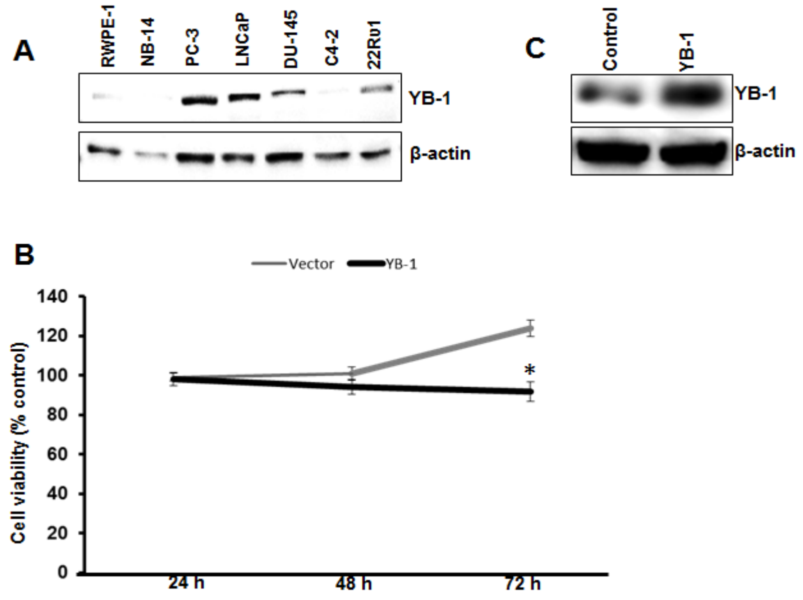

Supplementary Figure 2. YB-1 overexpression decreases cell proliferation. (A) YB-1 expression in various prostate cancer cell lines. (B)RWPE-1 cells were transfected with empty vector or YB-1 expression constructs and proliferation was measured by MTT conversion. Absorbance values are represented relative to time 0 (mean±s.d. of three independent biological replicates; *P<0.05). (C) RWPE-1 cells were transfected with empty vector or YB-1 expression constructs showed enhanced YB-1 expression.

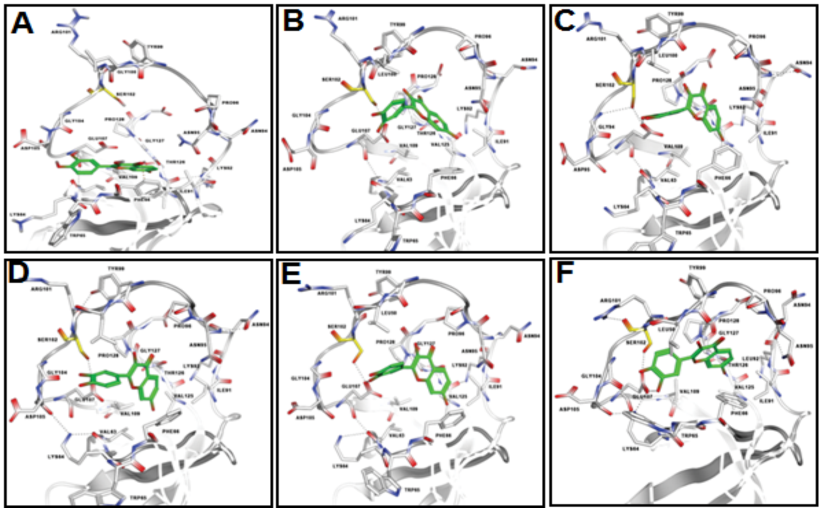

Supplementary Figure 3. Pose prediction of the energy-minimized average structures for the 300 ps MD of Fisetin/CSD YB-1 complex. Panels A-F: snapshots obtained for minimized structures generated from the 50 ps range period (50, 100, 150, 200, 250, and 300 ps). Important Ser102 residue in the flexible loop is highlighted in yellow.

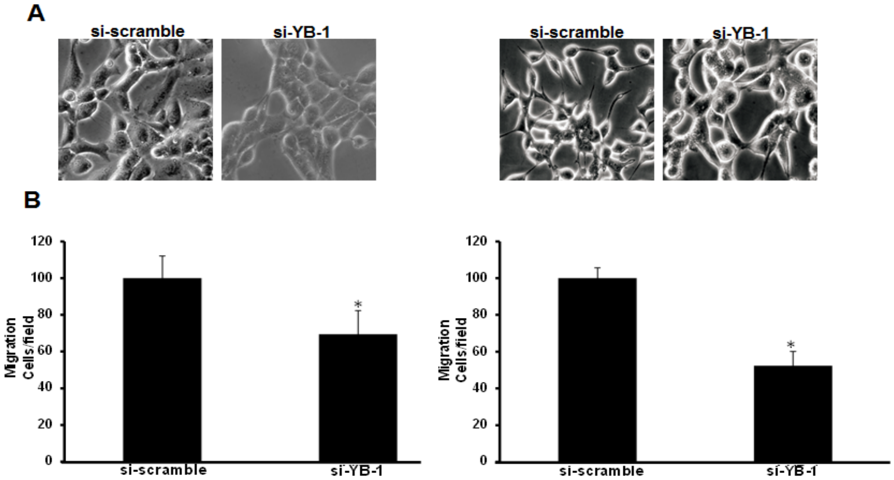

Supplementary Figure 4. YB-1 inhibition induces morphological changes in PCa cells. (A) DU145 and C4-2 cells were transfected with either scramble or YB-1 targeted siRNA and phase contrast images were taken using the same setting as describe in figure 2. (B) Histograms showing migration (mean ± SD) of DU145 and C4-2 cells in presence of siRNA YB-1. Mean cell numbers/field ± SD of experiments performed in triplicate is shown.
