# Supplementary material for: YB-1 expression promotes epithelial-to-mesenchymal transition in prostate cancer that is inhibited by a small molecule fisetin
Source: Oncotarget. 2014 Feb 19;5(9):2462–74. doi: 10.18632/oncotarget.1790 (PMC4058019; doi:10.18632/oncotarget.1790)
Supplement: Supplementary file 2 [file oncotarget-05-2462-s002.pdf]

Table S1. List of antibodies, dilutions, and suppliers used for immunoblotting (IB) and immunofluorescence (IF)

Antibody	Dilution	Supplier
β-actin	1:1000	Santa Cruz
E-cadherin	1:1000(IB);1:100(IF)	Cell Signaling
ZO-1	1:1000	Cell Signaling
N-cadherin	1:1000	Cell Signaling
Vimentin	1:1000(IB);1:100(IF)	Cell Signaling
Snail	1:1000	Cell Signaling
p-Akt^{ser473}	1:100(IP)	Cell Signaling
MTA-1	1:1000	Santa Cruz
Slug	1:200(IF)	Cell Signaling
Occludin	1:1000	Cell Signaling
YB-1	1:2000	Cell Signaling
pYB-1^{S102}	1:1000 (IB); 1:200 (IF)	Cell Signaling
Anti-HA	1:500	Sigma
